# A Case of Hodgkin's Lymphoma Diagnosed by Endobronchial Ultrasound-Guided Transbronchial Forceps Biopsies

**DOI:** 10.7759/cureus.44226

**Published:** 2023-08-27

**Authors:** Muzamil Khan, Khalil Diab

**Affiliations:** 1 Department of Pulmonary Medicine, The George Washington University, School of Medicine and Health Sciences, Washington DC, USA

**Keywords:** endobronchial ultrasound-guided transbronchial forceps biopsy, endobronchial ultrasound-guided transbronchial needle aspiration, mediastinal lymphadenopathy, hiv infection, ebus-tbna, hodgkin's lymphoma, ebus-tbfb

## Abstract

Endobronchial ultrasound (EBUS)-guided transbronchial needle aspiration (TBNA) has proven to be highly accurate in lung cancer diagnosis and staging. However, its efficacy in diagnosing lymphoma, especially Hodgkin’s lymphoma, remains controversial, mainly due to the need for larger biopsies for definitive diagnosis. This case study presents a 53-year-old HIV-positive man with a controlled viral load, who presented with a large left hilar mass and a left upper lobe nodule, both showing significant uptake on positron emission tomography scans. The patient underwent bronchoscopy with bronchoalveolar lavage, EBUS-TBNA using an Olympus™ Vizishot 2 needle (Center Valley, PA), and EBUS-guided transbronchial forceps biopsies (TBFB) of a left hilar lymph node using a 1.8 mm Boston Scientific™ forceps (Marlborough, MA). The EBUS-TBNA revealed granulomas, while the subsequent EBUS-guided TBFB revealed nodular lymphocyte-predominant Hodgkin's lymphoma. EBUS-TBFB may be a promising technique for obtaining larger tissue samples and enhancing diagnostic yield in cases of mediastinal lymphadenopathy with suspected lymphoma.

## Introduction

Endobronchial ultrasound (EBUS)-guided transbronchial needle aspiration (TBNA) is a widely used procedure and has been validated for the diagnosis and staging of lung cancer and the diagnosis of sarcoidosis. Despite its established role in lung cancer staging, its diagnostic accuracy in lymphoma remains a subject of debate due to certain disadvantages, such as lesser core tissue sampling and inferior negative predictive value [1]. This article highlights the importance of EBUS-guided transbronchial forceps biopsy (TBFB) in lymphoma diagnosis and discusses its role as a valuable adjunct to EBUS-TBNA in improving diagnostic accuracy.

## Case presentation

**Figure 1:**
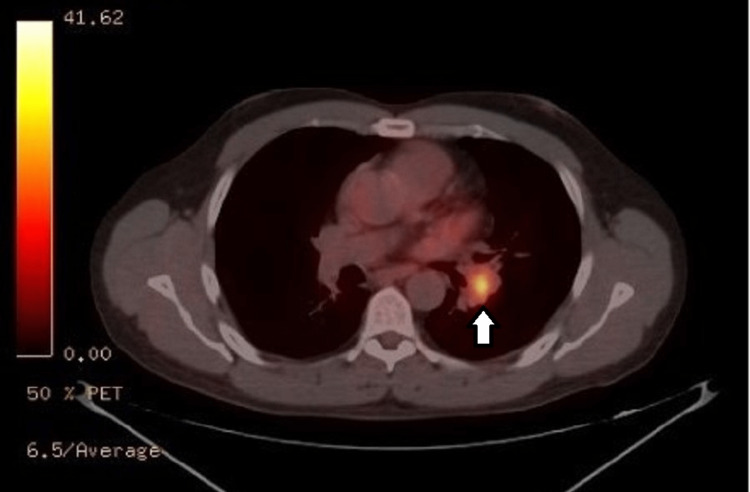
Positron emission tomography imaging of the left hilar mass, visually indicated by a distinct white arrow.

**Figure 2:**
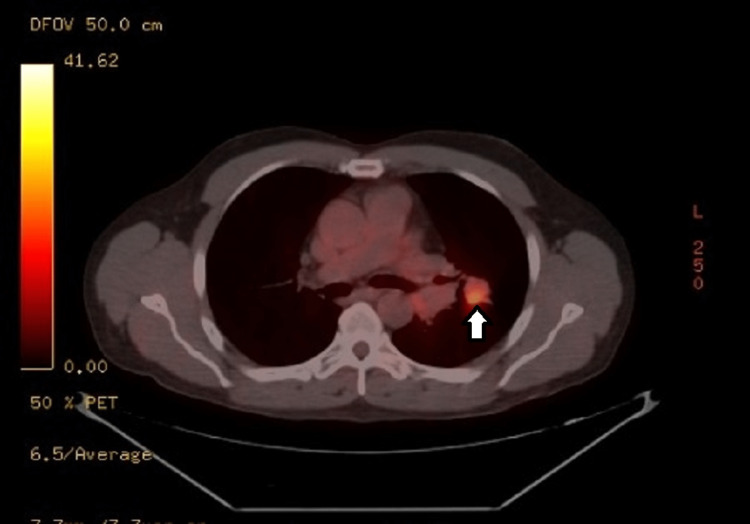
Positron emission tomography imaging of the left upper lobe. The left upper lobe nodule is visually indicated by a distinct white arrow.

A 53-year-old man with a medical history of obstructive sleep apnea, pulmonary nodule, and HIV infection presented with PET-positive left hilar mass (Figure 1) and left upper lobe (LUL) nodule (Figure 2).

**Figure 3:**
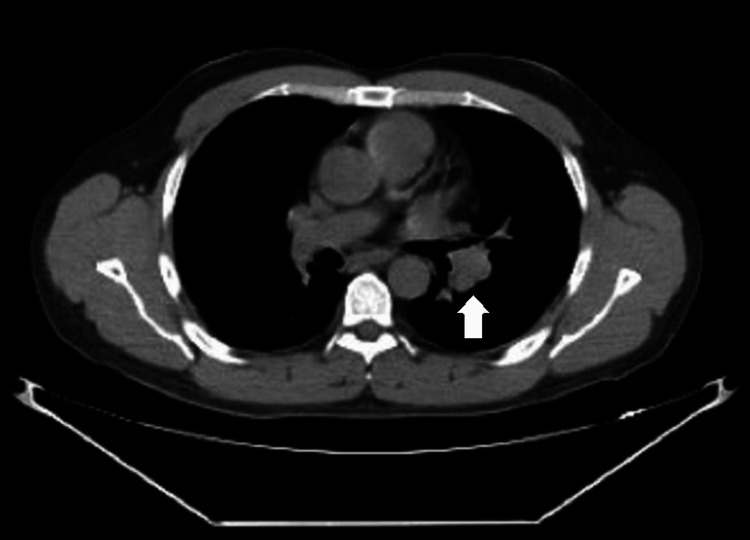
CT imaging of the left hilar mass, visually indicated by a distinct white arrow.

**Figure 4:**
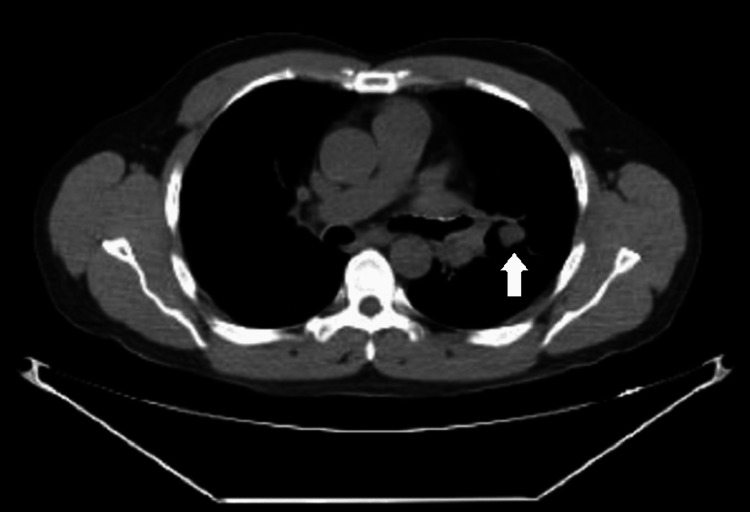
CT imaging of the left upper lobe. The left upper lobe nodule is visually indicated by a distinct white arrow.

The patient underwent bronchoscopy with bronchoalveolar lavage (BAL) of the LUL. The bronchoscope was introduced through an iGEL^TM^ (Intersurgical, Berkshire, UK) and wedged into the LUL bronchus. BAL was performed, and the fluid was sent for cultures, cytology, and antigen testing. EBUS-TBNA was then performed on the station 11L lymph node using a 22-gauge Vizishot 2 needle (Olympus, Center Valley, PA). A tract was formed by pushing the sheath in and dilating the area. Additionally, six transbronchial biopsy (TBBX) samples were obtained from the same lymph node under ultrasound guidance using 1.8 mm forceps. The lymph node TBNA revealed granulomatous inflammation. The lymph node biopsy using transbronchial forceps revealed nodular lymphocyte-predominant Hodgkin's lymphoma of intrathoracic lymph nodes, indicating HIV-associated Hodgkin's lymphoma. Figures 3, 4 illustrate CT imaging of the left hilar mass and the LUL nodule, respectively.

**Figure 5:**
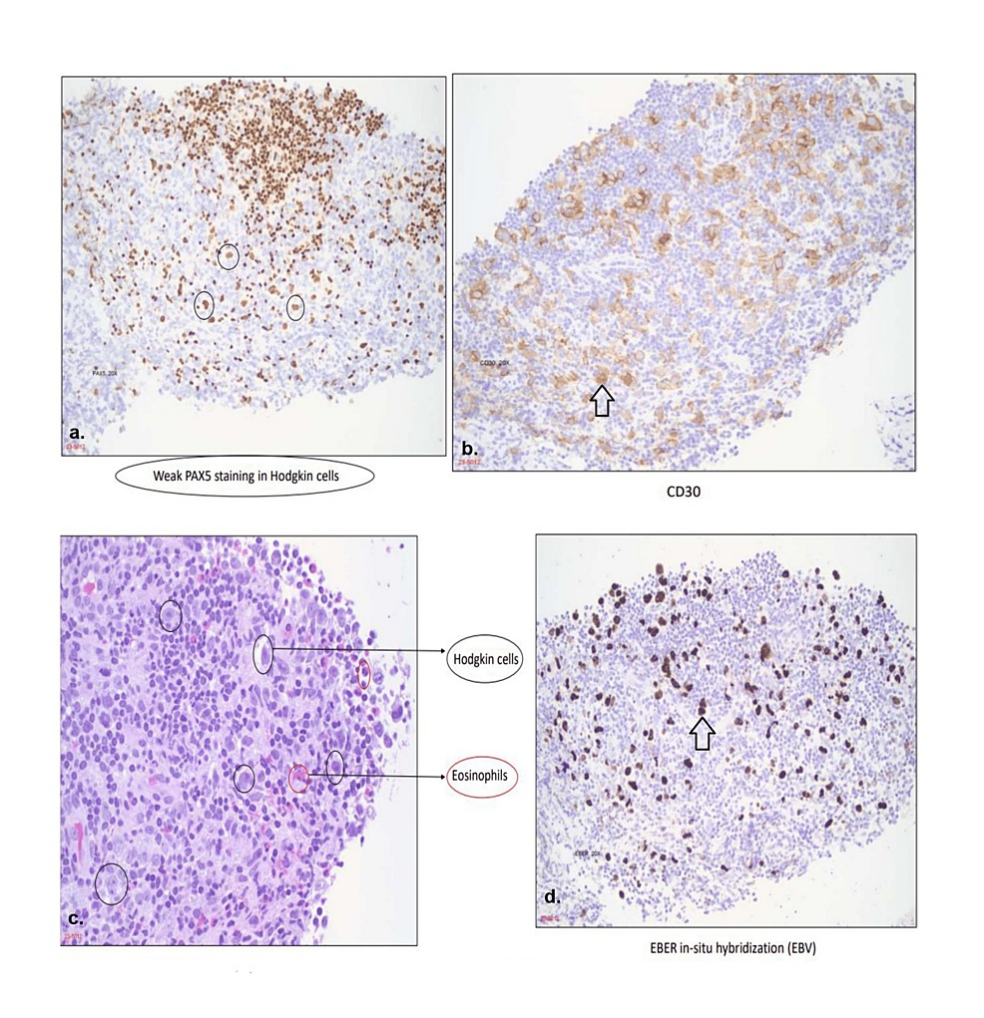
Histopathological images demonstrating the presence of Hodgkin's lymphoma. (a) Circles demonstrate weakly PAX5-stained Hodgkin cells. (b) Immunohistochemistry image in which arrow indicates CD30-positive cells. (c) Black circles indicate Hodgkin cells and red circles demonstrate the presence of eosinophils. (d) Arrow indicating EBV-positive cells. EBV: Epstein-Barr virus; EBER: Epstein-Barr encoding region.

During histopathological examination of the tissue samples, specific cells exhibited weak staining for PAX5, a critical transcription factor associated with B-cell development. This weakened staining pattern strongly suggests a decrease in the expression of B-cell markers, a hallmark characteristic of classic Hodgkin's lymphoma [2]. On immunohistochemistry, CD30-positive cells were identified. This revelation was accompanied by a distinctive light brown staining coloration, confirming the presence of cells that express CD30, a marker often associated with Hodgkin's lymphoma. Throughout the histopathological assessment, the presence of characteristic Hodgkin cells was also observed, highlighting the typical features of Hodgkin's lymphoma within the examined tissue [3]. Notably, eosinophils were also noted. Furthermore, an essential step in the evaluation process involved Epstein-Barr encoding region (EBER) hybridization, a procedure targeting Epstein-Barr virus (EBV) RNA detection [4]. During this procedure, a distinct brown staining was observed. This staining pattern indicates the presence of EBV RNA within the tissue sample [5]. Figure 5 illustrates the histopathological observations.

## Discussion

EBUS-TBFB provides a minimally invasive and accurate method for obtaining tissue samples from mediastinal lymph nodes, aiding in the diagnosis and staging of various pulmonary conditions, including Hodgkin's lymphoma. In scenarios where EBUS-TBNA fails to yield a diagnosis, as in our case, EBUS-TBFB can be a safe and effective alternative to improve diagnostic accuracy when performed in conjunction with EBUS-TBNA.

In a recent investigation, Pathak et al. studied a cohort of 30 patients, eight patients had a history of lymphoma, one patient had a history of squamous cell carcinoma, and one patient had a history of chronic lymphocytic leukemia with 25 of them undergoing EBUS-TBFB. The overall diagnostic yield of EBUS-TBFB was found to be 73%. However, when EBUS-TBFB was combined with EBUS-TBNA, the diagnostic yield significantly improved to 86% (26 out of 30 patients). While TBFB provided additional diagnostic information beyond TBNA in some cases, the study was not powered to thoroughly evaluate the diagnostic yield. These findings suggest that combining EBUS-TBFB with EBUS-TBNA may enhance diagnostic accuracy in the evaluation of patients with suspected lung pathology [6].

Mehta et al. aimed to assess the effectiveness and safety of a novel technique called EBUS-TBFB when conventional EBUS-TBNA with rapid on-site evaluation (ROSE) failed to provide a diagnosis. The study included 30 consecutive patients with enlarged mediastinal lymph nodes and negative EBUS-TBNA ROSE results. EBUS-TBFB successfully obtained adequate tissue samples in all cases where both EBUS-TBNA and EBUS-TBFB were performed. Particularly, in patients with non-diagnostic EBUS-TBNA ROSE, EBUS-TBFB yielded positive diagnostic results in eight out of 30 patients (27%), of which six were diagnosed with tuberculosis, one with aspergillosis, and one with Hodgkin’s lymphoma. For patients with a negative EBUS-TBNA ROSE, EBUS-TBFB proved to be a safe and valuable technique, contributing to the diagnostic yield. The findings indicate that EBUS-TBFB is a valuable addition to EBUS procedures, especially in benign causes of mediastinal lymph node enlargement [7].

In their study, Diab et al. compared the diagnostic yield of EBUS-TBNA and EBUS-TBFB individually with their combined approach in cases of mediastinal lymphadenopathy with unknown etiology. The combined technique provided a definitive diagnosis in 31 out of 35 cases (88.6%), accurately identifying both Hodgkin's and non-Hodgkin's lymphomas in 90% of cases without the need for further invasive procedures. Additionally, the combined approach successfully confirmed all cases of granulomatous inflammation. The study concludes that the safe and high diagnostic yield of the combination of EBUS-TBFB and EBUS-TBNA makes it particularly effective for diagnosing mediastinal adenopathy of unknown origin, especially in lymphoma cases. The larger samples obtained from TBFB also improved sensitivity in detecting granulomatous disease and provided specimens for clinical trials in malignancy cases when needle aspirates were insufficient [8].

Recently, there has been evidence supporting the high diagnostic success and safety of EBUS-TBFB for mediastinal lymph nodes. However, a rare technical issue was observed during the procedure, where a rupture of the steering band prevented the closure of forceps jaws in a subcarinal lymph node. This problem could lead to severe complications. The report highlights the importance of addressing such complications during transbronchial sampling of mediastinal lesions and presents a solution to this specific issue, along with other procedure-related complications reported in the literature [9].

In another study, combining bronchoscopic tissue forceps biopsy (BBX) and EBUS-TBNA improves diagnostic yield in lung lesion evaluation. In 115 patients, the combined approach provided a definitive diagnosis in 93% of cases, with 81% being malignant. Seventy-eight patients underwent BBX and EBUS-TBNA of the lymph node(s) only; malignancy was diagnosed in 63 (81%) patients; 28 (36%) had malignant cells in both specimen types, 19 (24%) had malignant cells only in the BBX, and 16 (21%) had malignant cells only in the EBUS-TBNA. In the remaining 15 patients who underwent BBX and EBUS-TBNA of the lymph nodes only, 10 (67%) were diagnosed with either granulomatous disease or evidence of infection, and five (33%) with malignancy on subsequent procedures. EBUS-TBNA was especially valuable when BBX results were negative, increasing diagnostic yield by 18% and assisting in tumor staging. Additionally, the combination allowed for more tissue sampling for immunohistochemistry and molecular testing, facilitating personalized management in a minimally invasive manner [1].

Our case demonstrates the importance of performing EBUS-TBFB in cases of mediastinal adenopathy of unknown etiology. Had we not performed EBUS-TBFB, EBUS-TBNA alone would have led us to a misdiagnosis of sarcoidosis in this case.

## Conclusions

EBUS-TBFB provides a minimally invasive and accurate method for obtaining tissue samples from mediastinal lymph nodes that may aid in the diagnosis and staging of various pulmonary conditions, including Hodgkin's lymphoma, non-Hodgkin’s lymphoma, and sarcoidosis. In scenarios where EBUS-TBNA fails to yield a diagnosis, EBUS-TBFB can be a safe and effective alternative to improve diagnostic accuracy when performed in conjunction with EBUS-TBNA. Further randomized controlled prospective trials are needed.
